# Supplementary material for: Linear antibody epitope prediction using AlphaFold2
Source: eLife. 2026 Sep 18;13:RP98369. doi: 10.7554/eLife.98369 (PMC13588620; doi:10.7554/eLife.98369)
Supplement: Supplementary file 2 [file elife-98369-supp2.pdf]

```
Kabat numbering     1--------10--------20--------30--------40--------50-------
MYC               -MEVKLVESGGDLVKPGGSLKLSCAASGFTFSHYGMSWVRQTPDKRLEWVATIG--SRGT
MYC-2E2           MAEVQLVESGGDLVKPGGSLKLSCAASGFTFSHYGMSWVRQTPDKRLEWVATIG--SRGT
MYC-15F11         -MEVKLVESGGGLVKPGGSLKLSCAASGFTFSHYGMSWVRQTPEKRLEWVATIG--SRGT
mBG17             MAEVKLEESGGGLVQPGGSMKFSCVASGFTFSDYWMNWVRQSPDKGLEWVAEIRLKSNNY
mBG17-2E2         MAEVQLVESGGDLVKPGGSLKLSCAASGFTFSDYWMNWVRQTPDKRLEWVAEIRLKSNNY
mBG17-15F11       MAEVKLVESGGGLVKPGGSLKLSCAASGFTFSDYWMNWVRQTPEKRLEWVAEIRLKSNNY
HA-scFv           MAEVKLVESGGDLVKPGGSLKLSCAASGFTFSSYGMSWVRQTPDKRLEWVATISRG--GS
HA-2E2            MAEVQLVESGGDLVKPGGSLKLSCAASGFTFSSYGMSWVRQTPDKRLEWVATISRG--GS
HA-15F11          -MEVKLVESGGGLVKPGGSLKLSCAASGFTFSSYGMSWVRQTPEKRLEWVATISRG--GS

Kabat numbering   ---------65---70--------80-----------90---95---------------102
MYC               YTHYPDSVKGRFTISRDNDKNALYLQMNSLKSEDTAMYYCARRSEFYYYGNTYYYSAMDY
MYC-2E2           YTHYPDSVKGRFTISRDNAKNTLYLQMSSLKSEDTAMYYCARRSEFYYYGNTYYYSAMDY
MYC-15F11         YTHYPDSVKGRFTISRDNAKNTLYLQMSSLRSEDTAIYYCARRSEFYYYGNTYYYSAMDY
mBG17             ATHYAASVKGRFTISRDDSKSSVYLQMNNLRAEDSGIYYCTRS-------------AMDY
mBG17-2E2         ATHYAASVKGRFTISRDNAKNTLYLQMSSLKSEDTAMYYCARS-------------AMDY
mBG17-15F11       ATHYAASVKGRFTISRDNAKNTLYLQMSSLRSEDTAIYYCARS-------------AMDY
HA-scFv           YTYYPDSVKGRFTISRDNAKNTLYLQMSSLKSEDTAMYYCARRET-------YDEKGFAY
HA-2E2            YTYYPDSVKGRFTISRDNAKNTLYLQMSSLKSEDTAMYYCARRET-------YDEKGFAY
HA-15F11          YTYYPDSVKGRFTISRDNAKNTLYLQMSSLRSEDTAIYYCARRET-------YDEKGFAY

                  -------110-              1--------10-----------24---------
MYC               WGQGASVTVSSGGGGSGGGGSGGGGSDIVLTQSPASLAVSLGQRATISCRASESVDNYG-
MYC-2E2           WGQGTSVTVSSGGGGSGGGGSGGGGSDIVLTQSPASLAVSLGQRATISCRASESVDNYG-
MYC-15F11         WGQGTTLTVSSGGGGSGGGGSGGGGSDIVLTQSPASLTVSLGQRATISCRASESVDNYG-
mBG17             WGQGTSVTVSSGGGGSGGGGSGGGGSDIVMSQSPSSLAVSVGEKITMSCKSSQSLLYTSD
mBG17-2E2         WGQGTSVTVSSGGGGSGGGGSGGGGSDIVLTQSPASLAVSLGQRATISCKSSQSLLYTSD
mBG17-15F11       WGQGTTLTVSSGGGGSGGGGSGGGGSDIVLTQSPASLTVSLGQRATISCKSSQSLLYTSD
HA-scFv           WGQGTTVTVSSGGGGSGGGGSGGGGSDIELTQSPSSLTVTAGEKVTMSCKSSQSLLNSGN
HA-2E2            WGQGTSVTVSSGGGGSGGGGSGGGGSDIVLTQSPASLAVSLGQRATISCKSSQSLLNSGN
HA-15F11          WGQGTTLTVSSGGGGSGGGGSGGGGSDIVLTQSPASLTVSLGQRATISCKSSQSLLNSGN

                  -----34----40--------50--------60--------70--------80-------
MYC               -FSFMNWFQQKPGQPPKLLIYAISNRGSGVPARFSGSGSGTDFSLNIHPVEEDDPAMYFC
MYC-2E2           -FSFMNWYQQKPGQPPKLLIYAISNRGSGIPARFSGSGSGTDFTLNIHPVEEEDAATYYC
MYC-15F11         -FSFMNWYQQKPGQPPKLLIYAISNRGSGIPARFSGSGSGTDFTLNIHPVEEEDAATYYC
mBG17             QKNYLAWFQQKPGQSPKLLIFWASTRDSGVPDRFTGSGSGTDFTLTISSVKAEDLAVYYC
mBG17-2E2         QKNYLAWYQQKPGQPPKLLIYWASTRDSGIPARFSGSGSGTDFTLNIHPVEEEDAATYYC
mBG17-15F11       QKNYLAWYQQKPGQPPKLLIYWASTRDSGIPARFSGSGSGTDFTLNIHPVEEEDAATYYC
HA-scFv           QKNYLTWYQQKPGQPPKLLIYWASTRESGVPDRFTGSGSGRDFTLTISSVQAEDLAVYYC
HA-2E2            QKNYLTWYQQKPGQPPKLLIYWASTRESGIPARFSGSGSGTDFTLNIHPVEEEDAATYYC
HA-15F11          QKNYLTWYQQKPGQPPKLLIYWASTRESGIPARFSGSGSGTDFTLNIHPVEEEDAATYYC

                  -90--------100----
MYC               QQTKEVPWTFGGGTKLEI
MYC-2E2           QQTKEVPWTFGGGTKLEI
MYC-15F11         QQTKEVPWTFGAGTKLEI
mBG17             QQFYNYPRTFGGGTKLEI
mBG17-2E2         QQFYNYPRTFGGGTKLEI
mBG17-15F11       QQFYNYPRTFGAGTKLEI
HA-scFv           QNDNSHPLTFGAGTKLEL
HA-2E2            QNDNSHPLTFGGGTKLEI
HA-15F11          QNDNSHPLTFGAGTKLEI
```

Legend: Heavy chain loops | linker | Light chain loops
