# Supplementary material for: Linear antibody epitope prediction using AlphaFold2
Source: eLife. 2026 Sep 18;13:RP98369. doi: 10.7554/eLife.98369 (PMC13588620; doi:10.7554/eLife.98369)
Supplement: Supplementary file 3 [file elife-98369-supp1.docx]

**Supplemental Table 1A**

>mBG17 scFv

MAEVKLEESGGGLVQPGGSMKFSCVASGFTFSDYWMNWVRQSPDKGLEWVAEIRLKSNNYATHYAASVKGRFTISRDDSKSSVYLQMNNLRAEDSGIYYCTRSAMDYWGQGTSVTVSSGGGGSGGGGSGGGGSDIVMSQSPSSLAVSVGEKITMSCKSSQSLLYTSDQKNYLAWFQQKPGQSPKLLIFWASTRDSGVPDRFTGSGSGTDFTLTISSVKAEDLAVYYCQQFYNYPRTFGGGTKLEI

>mBG17-15F11

MAEVKLVESGGGLVKPGGSLKLSCAASGFTFSDYWMNWVRQTPEKRLEWVAEIRLKSNNYATHYAASVKGRFTISRDNAKNTLYLQMSSLRSEDTAIYYCARSAMDYWGQGTTLTVSSGGGGSGGGGSGGGGSDIVLTQSPASLTVSLGQRATISCKSSQSLLYTSDQKNYLAWYQQKPGQPPKLLIYWASTRDSGIPARFSGSGSGTDFTLNIHPVEEEDAATYYCQQFYNYPRTFGAGTKLEI

>mBG17-2E2

MAEVQLVESGGDLVKPGGSLKLSCAASGFTFSDYWMNWVRQTPDKRLEWVAEIRLKSNNYATHYAASVKGRFTISRDNAKNTLYLQMSSLKSEDTAMYYCARSAMDYWGQGTSVTVSSGGGGSGGGGSGGGGSDIVLTQSPASLAVSLGQRATISCKSSQSLLYTSDQKNYLAWYQQKPGQPPKLLIYWASTRDSGIPARFSGSGSGTDFTLNIHPVEEEDAATYYCQQFYNYPRTFGGGTKLEI

>mBG17 Fab VH:VL

MYLGLNCVFIVFLLKGVQSEVKLEESGGGLVQPGGSMKFSCVASGFTFSDYWMNWVRQSPDKGLEWVAEIRLKSNNYATHYAASVKGRFTISRDDSKSSVYLQMNNLRAEDSGIYYCTRSAMDYWGQGTSVTVSS:MDSQAQVLMLLLLWVSGTCGDIVMSQSPSSLAVSVGEKITMSCKSSQSLLYTSDQKNYLAWFQQKPGQSPKLLIFWASTRDSGVPDRFTGSGS

>mBG17 epitope

DDFSKQLQQS

>mBG17 target protein sequence – SARS CoV-2 Nucleocapsid protein

MSDNGPQNQRNAPRITFGGPSDSTGSNQNGERSGARSKQRRPQGLPNNTASWFTALTQHGKEDLKFPRGQGVPINTNSSPDDQIGYYRRATRRIRGGDGKMKDLSPRWYFYYLGTGPEAGLPYGANKDGIIWVATEGALNTPKDHIGTRNPANNAAIVLQLPQGTTLPKGFYAEGSRGGSQASSRSSSRSRNSSRNSTPGSSRGTSPARMAGNGGDAALALLLLDRLNQLESKMSGKGQQQQGQTVTKKSAAEASKKPRQKRTATKAYNVTQAFGRRGPEQTQGNFGDQELIRQGTDYKHWPQIAQFAPSASAFFGMSRIGMEVTPSGTWLTYTGAIKLDDKDPNFKDQVILLNKHIDAYKTFPPTEPKKDKKKKADETQALPQRQKKQQTVTLLPAADLDDFSKQLQQSMSSADSTQA

>HA scFv

MAEVKLVESGGDLVKPGGSLKLSCAASGFTFSSYGMSWVRQTPDKRLEWVATISRGGSYTYYPDSVKGRFTISRDNAKNTLYLQMSSLKSEDTAMYYCARRETYDEKGFAYWGQGTTVTVSSGGGGSGGGGSGGGGSDIELTQSPSSLTVTAGEKVTMSCKSSQSLLNSGNQKNYLTWYQQKPGQPPKLLIYWASTRESGVPDRFTGSGSGRDFTLTISSVQAEDLAVYYCQNDNSHPLTFGAGTKLEL

>HA-15F11

MEVKLVESGGGLVKPGGSLKLSCAASGFTFSSYGMSWVRQTPEKRLEWVATISRGGSYTYYPDSVKGRFTISRDNAKNTLYLQMSSLRSEDTAIYYCARRETYDEKGFAYWGQGTTLTVSSGGGGSGGGGSGGGGSDIVLTQSPASLTVSLGQRATISCKSSQSLLNSGNQKNYLTWYQQKPGQPPKLLIYWASTRESGIPARFSGSGSGTDFTLNIHPVEEEDAATYYCQNDNSHPLTFGAGTKLEI

>HA-2E2

MAEVQLVESGGDLVKPGGSLKLSCAASGFTFSSYGMSWVRQTPDKRLEWVATISRGGSYTYYPDSVKGRFTISRDNAKNTLYLQMSSLKSEDTAMYYCARRETYDEKGFAYWGQGTSVTVSSGGGGSGGGGSGGGGSDIVLTQSPASLAVSLGQRATISCKSSQSLLNSGNQKNYLTWYQQKPGQPPKLLIYWASTRESGIPARFSGSGSGTDFTLNIHPVEEEDAATYYCQNDNSHPLTFGGGTKLEI

>HA target protein sequence – influenza hemmaglutanin A

MKTIIALSYILCLVSAQKLPGSENRTATLCLGHHAVQNGTLVKTITNDQIEVTNATELVQSSSTGRICDNPHRVLDGRDCTLIDALLGDPHCDSFQNKEWDLFIERSKAYSNCYPYDVPDYASLRSLVASSGTLEFTTEGFDWTGVTQNGTSYSCKRGSANSFFSRLNWLHKLNYKYPAQNVTMPNDDKFDKLYIWGVHHPSTDNDQTSLYVQTSGRVTVSTKRSQQTVVPDIGSRPWVRGISSRISIHWTIVKPGDILLINSTGNLIAPRGYFKIRNGKSSIMKSDALIGNCNSECITPNGSIPNDKPFQNVNRITYGDCPRYVKQSTLKLATGMRNVPEKQTRGIFGAIAGFIENGWEGMVDGWYGFRHRNSEGTGQAADLKSTQAAIDQINGKLNRLIKKTNEKFHQIEKEFSEVEGRIQDLEKYVEDTKVDLWSYNAELLVALENQHTIDLTDSEMNKLFERTRKQLRENAEDMGNGCFKIYHRCDNACIGSIRNGTYNHNVYRDEALNNRFKIKGVELKSGYKDWILWISFAISCFLLCVGLMGLIMWTCQKGNIRCIRCNICH

>HA epitope

YPYDVPDYA

>Myc scFv

MEVKLVESGGDLVKPGGSLKLSCAASGFTFSHYGMSWVRQTPDKRLEWVATIGSRGTYTHYPDSVKGRFTISRDNDKNALYLQMNSLKSEDTAMYYCARRSEFYYYGNTYYYSAMDYWGQGASVTVSSGGGGSGGGGSGGGGSDIVLTQSPASLAVSLGQRATISCRASESVDNYGFSFMNWFQQKPGQPPKLLIYAISNRGSGVPARFSGSGSGTDFSLNIHPVEEDDPAMYFCQQTKEVPWTFGGGTKLEI

>Myc-15F11

MEVKLVESGGGLVKPGGSLKLSCAASGFTFSHYGMSWVRQTPEKRLEWVATIGSRGTYTHYPDSVKGRFTISRDNAKNTLYLQMSSLRSEDTAIYYCARRSEFYYYGNTYYYSAMDYWGQGTTLTVSSGGGGSGGGGSGGGGSDIVLTQSPASLTVSLGQRATISCRASESVDNYGFSFMNWYQQKPGQPPKLLIYAISNRGSGIPARFSGSGSGTDFTLNIHPVEEEDAATYYCQQTKEVPWTFGAGTKLEI

>Myc-2E2

MAEVQLVESGGDLVKPGGSLKLSCAASGFTFSHYGMSWVRQTPDKRLEWVATIGSRGTYTHYPDSVKGRFTISRDNAKNTLYLQMSSLKSEDTAMYYCARRSEFYYYGNTYYYSAMDYWGQGTSVTVSSGGGGSGGGGSGGGGSDIVLTQSPASLAVSLGQRATISCRASESVDNYGFSFMNWYQQKPGQPPKLLIYAISNRGSGIPARFSGSGSGTDFTLNIHPVEEEDAATYYCQQTKEVPWTFGGGTKLEI

>Myc target protein sequence

MDFFRVVENQPPATMPLNVSFTNRNYDLDYDSVQPYFYCDEEENFYQQQQQSELQPPAPSEDIWKKFELLPTPPLSPSRRSGLCSPSYVAVTPFSLRGDNDGGGGSFSTADQLEMVTELLGGDMVNQSFICDPDDETFIKNIIIQDCMWSGFSAAAKLVSEKLASYQAARKDSGSPNPARGHSVCSTSSLYLQDLSAAASECIDPSVVFPYPLNDSSSPKSCASQDSSAFSPSSDSLLSSTESSPQGSPEPLVLHEETPPTTSSDSEEEQEDEEEIDVVSVEKRQAPGKRSESGSPSAGGHSKPPHSPLVLKRCHVSTHQHNYAAPPSTRKDYPAAKRVKLDSVRVLRQISNNRKCTSPRSSDTEENVKRRTHNVLERQRRNELKRSFFALRDQIPELENNEKAPKVVILKKATAYILSVQAEEQKLISEEDLLRKRREQLKHKLEQLRNSCA

>Myc epitope

EQKLISEEDL
