# Supplementary material for: Linear antibody epitope prediction using AlphaFold2
Source: eLife. 2026 Sep 18;13:RP98369. doi: 10.7554/eLife.98369 (PMC13588620; doi:10.7554/eLife.98369)
Supplement: Peer review history

# Editor report: eLife Assessment

**DOI:** 10.7554/eLife.98369.3.sa0

**Authors:** Volker Dötsch

**Affiliations:** Goethe University Frankfurt Germany

**Keywords:** Incomplete, Useful

In this article, the authors describe a new AlphaFold2 pipeline called PabFold that can represent a **useful** tool for identifying linear antibody epitopes (B-cell epitopes) for different antigens. This information can be used in the selection of different reagents in competitive ELISA assays, which can save time and reduce costs. Because several questions about the work remain, the study is currently **incomplete**.

# Referee report: Reviewer #1 (Public Review)

**DOI:** 10.7554/eLife.98369.3.sa1

Summary:

In this manuscript, "PAbFold: Linear Antibody Epitope Prediction using AlphaFold2", the authors generate a python wrapper for the screening of antibody-peptide interactions using AlphaFold, and test the performance of AlphaFold on 3 antibody-peptide complexes. In line with previous observations regarding the ability of AlphaFold to predict antibody structures and antigen binding, the results are mixed. While the authors are able to use AlphaFold to identify and experimentally validate a previously characterized broad binding epitope with impressive precision, they are unable to consistently identify the proper binding registers for their control [Myc-tag, HA-tag] peptides. Further, it appears that the reproducibility and generality of these results are low, with new versions of AlphaFold negatively impacting the predictive power. However, if this reproducibility issue is solved, and the test set is greatly increased, this manuscript could contribute strongly towards our ability to predict antibody-antigen interactions.

Strengths:

Due to the high significance, but difficulty, of the prediction of antibody-antigen interactions, any attempts to break down these predictions into more tractable problems should be applauded. The authors' approach of focusing on linear epitopes (peptides) is clever, reducing some of the complexities inherent to antibody binding. Further, the ability of AlphaFold to narrow down a previously broadly identified experimental epitope is impressive. The subsequent experimental validation of this more precisely identified epitope makes for a nice data point in the assessment of AlphaFold's ability to predict antibody-antigen interactions.

Weaknesses:

Without a larger set of test antibody-peptide interactions, it is unclear whether or not AlphaFold can precisely identify the binding register of a given antibody to a given peptide antigen. Even within the small test set of 3 antibody-peptide complexes, performance is variable and depends upon the scFv scaffold used for unclear reasons. Lastly, the apparent poor reproducibility is concerning, and it is not clear why the results should rely so strongly on which multi-sequence alignment (MSA) version is used, when neither the antibody CDR loops nor the peptide are likely to strongly rely on these MSAs for contact prediction.

Major Point-by-Point Comments:

(1) The central concern for this manuscript is the apparent lack of reproducibility. The way the authors discuss the issue (lines 523-554) it sounds as though they are unable to reproduce their initial results (which are reported in the main text), even when previous versions of AlphaFold2 are used. If this is the case, it does not seem that AlphaFold can be a reliable tool for predicting antibody-peptide interactions.

(2) Aside from the fundamental issue of reproducibility, the number of validating tests is insufficient to assess the ability of AlphaFold to predict antibody-peptide interactions. Given the authors' use of AlphaFold to identify antibody binding to a linear epitope within a whole protein (in the mBG17:SARS-Cov-2 nucleocapsid protein interaction), they should expand their test set well beyond Myc- and HA-tags using antibody-antigen interactions from existing large structural databases.

(3) As discussed in lines 358-361, the authors are unsure if their primary control tests (antibody binding to Myc-tag and HA-tag) are included in the training data. Lines 324-330 suggest that even if the peptides are not included in the AlphaFold training data because they contain fewer than 10 amino acids, the antibody structures may very well be included, with an obvious "void" that would be best filled by a peptide. The authors must confirm that their tests are not included in the AlphaFold training data, or re-run the analysis with these templates removed.

(4) The ability of AlphaFold to refine the linear epitope of antibody mBG17 is quite impressive and robust to the reproducibility issues the authors have run into. However, Figure 4 seems to suggest that the target epitope adopts an alpha-helical structure. This may be why the score is so high and the prediction is so robust. It would be very useful to see along with the pLDDT by residue plots a structure prediction by residue plot. This would help to see if the high confidence pLDDT is coming more from confidence in the docking of the peptide or confidence in the structure of the peptide.

(5) Related to the above comment, pLDDT is insufficient as a metric for assessing antibody-antigen interactions. There is a chance (as is nicely shown in Figure S3C) that AlphaFold can be confident and wrong. Here we see two orange-yellow dots (fairly high confidence) that place the peptide COM far from the true binding region. While running the recommended larger validation above, the authors should also include a peptide RMSD or COM distance metric, to show that the peptide identity is confident, and the peptide placement is roughly correct. These predictions are not nearly as valuable if AlphaFold is getting the right answer for the wrong reasons (i.e. high pLDDT but peptide binding to a non-CDR loop region). Eventual users of the software will likely want to make point mutations or perturb the binding regions identified by the structural predictions (as the authors do in Figure 4).

Comments on revisions:

I have read the author's responses and the revised manuscript. The authors did not sufficiently address my comments, nor the fundamental issue with the manuscript.

By the authors' own admission, many of the results presented in the current version of the manuscript cannot be reproduced without relying on locally saved MSAs. In other words, there is almost no evidence presented that this pipeline will predict antibody-antigen interactions using currently publicly available software. This manuscript is reduced to essentially a case study (N=1) in how one might go about making such predictions coupled with pretty good experimental evidence backing up this singular prediction.

# Referee report: Reviewer #2 (Public Review)

**DOI:** 10.7554/eLife.98369.3.sa2

Summary:

The authors showed the applicability and usefulness of a new AlphaFold2 pipeline called PabFold, which can predict linear antibody epitopes (B-cell epitopes) that can be helpful for the selection of reagents to be applied in competitive ELISA assay.

Strengths:

The authors showed the accuracy of the pipeline to identify correctly the binding epitope for three different antibody-antigen systems (Myc, HA, and Sars-Cov2 nucleocapsid protein). The design of scFvs from Fab of the three antibodies to speed up the analysis time is extremely interesting.

Weaknesses:

The article justifies correctly the findings and no great weaknesses are present. However, it could be useful for a broader audience to show in detail how pLDDT was calculated for both Simple-Max approach (per residue-pLDDT) and Consensus analysis (average pLDDT for each peptide), with associated equations.

Comments on revisions:

I have read the author's responses to my comments and the revised paper. They addressed the minor comments and concerns. However, I agree with Reviewer #1 that these findings cannot be reproduced without local MSAs and this is a major issue.

# Author comment: Author response

**DOI:** 10.7554/eLife.98369.3.sa3

**Authors:** Jacob DeRoo, James S Terry, Ning Zhao, Timothy J Stasevich, Christopher Snow, Brian J Geiss

**Affiliations:** Colorado State University Fort Collins United States; Colorado State Univeristy Fort Collins United States; University of Colorado - Anschutz Medical Campus Aurora United States; Colorado State University Fort Collins United States; Colorado State University Fort Collins United States; Colorado State University Fort Collins United States

The following is the authors’ response to the original reviews.

**Reviewer 1 (Public Comments):**

(1) The central concern for this manuscript is the apparent lack of reproducibility. The way the authors discuss the issue (lines 523-554) it sounds as though they are unable to reproduce their initial results (which are reported in the main text), even when previous versions of AlphaFold2 are used. If this is the case, it does not seem that AlphaFold can be a reliable tool for predicting antibody-peptide interactions.

The driving point behind the multiple sequence alignment (MSA) discussion was indeed to point out that AlphaFold2 (AF2) performance when predicting scFv:peptide complexes is highly dependent upon the MSA, but that is a function of MSA generation algorithm (MMseqs2, HHbiltz, jackhmmer, hhsearch, kalign, etc) and sequence databases, and less an intrinsic function of AF2. It is important to report MSA-dependent performance precisely because this results in changing capabilities with respect to peptide prediction.

Performance also significantly varies with the target peptide and scFv framework changes. By reporting the varying success rates (as a function of MSA, peptide target, and framework changes) we aim to help future researchers craft modified algorithms that can achieve increased reliability at protein-peptide binding predictions. Ultimately, tracking down how MSA generation details vary results (especially when the MSA’s are hundreds long) is significantly outside the scope of this paper. Our goal for this paper was to show a general method for identification of linear antibody epitopes using only sequence information, and future work by us or others should focus on optimization of the process.

(2) Aside from the fundamental issue of reproducibility, the number of validating tests is insufficient to assess the ability of AlphaFold to predict antibody-peptide interactions. Given the authors' use of AlphaFold to identify antibody binding to a linear epitope within a whole protein (in the mBG17:SARS-Cov-2 nucleocapsid protein interaction), they should expand their test set well beyond Myc- and HA-tags using antibody-antigen interactions from existing large structural databases.

Performing the calculations at the scale that the reviewer is requesting is not feasible at this time. We showed in this manuscript that we were able to predict 3 of 3 epitopes, including one antigen and antibody pair that have not been deposited into the PDB with no homologs. While we feel that an N=3 is acceptable to introduce this method to the scientific community, we will consider adding more examples of success and failure in the future to optimize and refine the method as computational resources become available. Notably, future efforts that attempt high-throughput predictions of this class using existing databases should take particular care to avoid contamination.

(3) As discussed in lines 358-361, the authors are unsure if their primary control tests (antibody binding to Myc-tag and HA-tag) are included in the training data. Lines 324-330 suggest that even if the peptides are not included in the AlphaFold training data because they contain fewer than 10 amino acids, the antibody structures may very well be included, with an obvious "void" that would be best filled by a peptide. The authors must confirm that their tests are not included in the AlphaFold training data, or re-run the analysis with these templates removed.

First, we address the simpler question of templates.

The reruns of AF2 with the local 2022 rebuild, the most reproducible method used with results most on par with the MMSEQS server in the Fall of 2022, were run without templates. This is because the MSA was generated locally; no templates were matched and generated locally. The only information passed then was the locally generated MSA, and the fasta sequence of the unchanging scFv and the dynamic epitope sequence. Because of how well this performed despite the absence of templates, we can confidently say the inclusion of the template flag is not significant with respect to how universally accurately PAbFold can identify the correct epitope.

Second, we can partially address the question of whether the AlphaFold models had access to models suitable, in theory, for “memorization” of pertinent structural details.

With respect to tracking the exact role and inclusion of specific PDB entries, the AF2 paper provides the following:

“Structures from the PDB were used for training and as templates (https://www.wwpdb.org/ftp/pdb-ftp-sites; for the associated sequence data and 40% sequence clustering see also https://ftp.wwpdb.org/pub/pdb/derived_data/ and https://cdn.rcsb.org/resources/sequence/clusters/bc-40.out). Training used a version of the PDB downloaded 28 August 2019, while the CASP14 template search used a version downloaded 14 May 2020. The template search also used the PDB70 database, downloaded 13 May 2020 (https://wwwuser.gwdg.de/~compbiol/data/hhsuite/databases/hhsuite_dbs/).”

Three of these links are dead. As such, it is difficult to definitively assess the role of any particular PDB entry with respect to AF2 training/testing, nor what impact homologous training structures given the very large number of immunoglobin structures in the training set. That said, we can summarize information for the potentially relevant PDB entries (l 2or9, which is shown in Fig. 1 and 1frg), and believe it is most conservative to assume that each such entry was within the training set.

PDB entry 2or9 (released 2008): the anti-c-myc antibody 9E10 Fab fragment in complex with an 11-amino acid synthetic epitope: EQKLISEEDLN. This crystal structure is also noteworthy for featuring a binding mode where the peptide is pinned between two Fab. The apo structure (2orb) is also in the database but lacks the peptide and a resolved structure for CDR H3.

PDB entry 1a93 (released 1998): a c-Myc-Max leucine zipper structure, where the c-Myc epitope (in a 34-amino acid protein) adopts an alpha helical conformation completely different from the epitope captured in entry 2or9.

PDB entries 5xcs and 5xcu (released 2017): engineered Fv-clasps (scFv alternatives) in complex with the 9-amino acid synthetic HA epitope: YPYDVPDYA.

PDB entry 1frg (released 1994): anti-HA peptide Fab in complex with HA epitope subset Ace-DVPDYASL-NH2.

Since the 2or9 entry has our target epitope (10 aa) embedded within an 11aa sequence, we have revised this line in the manuscript:

The AlphaFold2 training set was reported to exclude chains of less than 10, which would eliminate the myc and HA epitope peptides. => The AlphaFold2 training set was reported to exclude chains of less than 10, which would eliminate the HA epitope peptide from potential training PDB entries such as 5xcs or 5xcu”

It is important to note that we obtained the best prediction performance for the scFv:peptide pair that had no pertinent PDB entries (mBG17). Specifically, doing a Protein Blast against the PDB using the mBG17 scFv revealed diverse homologs, but a maximum sequence identity of 89.8% for the heavy chain (to an unrelated antibody) and 93.8% for the light chain (to an unrelated antibody). Additionally, while it is possible that the AF2 models might have learned from the complex in pdb entry 2or9, Supplemental Figure 3 shows how often the peptide is “misplaced”, and the performance does not exceed the performance for mBG17.

(4) The ability of AlphaFold to refine the linear epitope of antibody mBG17 is quite impressive and robust to the reproducibility issues the authors have run into. However, Figure 4 seems to suggest that the target epitope adopts an alpha-helical structure. This may be why the score is so high and the prediction is so robust. It would be very useful to see along with the pLDDT by residue plots a structure prediction by residue plot. This would help to see if the high confidence pLDDT is coming more from confidence in the docking of the peptide or confidence in the structure of the peptide.

The reviewer is correct that target mBG17 epitope adopts an alpha helical conformation, and we concur that this likely contributes to the more reliable structure prediction performance. When we predict the structure of the epitope alone without the mBG17 scFv, AF2 confidently predicts an alpha helix with an average pLDDT of 88.2 (ranging from 74.6 to 94.4).

**Author response image 1.** The AF2 prediction for the mBG17 epitope by itself.

However, as one interesting point of comparison, a 10 a.a. poly-alanine peptide is also consistently folded into an alpha-helical coil by AF2. The A_10_ peptide is also predicted to bind among the traditional scFv CDR loops, but the pLDDT scores are very poor (Supplemental Figure 5J). We also observed the opposite case; when a peptide has a very unstructured region in the binding domain but is nonetheless still be placed confidently, as seen in Supplemental Figure 3 C&D. Therefore, while we suspect peptides with strong alpha helical propensity are more *likely* to be accurately predicted, the data suggests that that alpha helix adoption is neither necessary nor sufficient to reach a confident prediction.

(5) Related to the above comment, pLDDT is insufficient as a metric for assessing antibody antigen interactions. There is a chance (as is nicely shown in Figure S3C) that AlphaFold can be confident and wrong. Here we see two orange-yellow dots (fairly high confidence) that place the peptide COM far from the true binding region. While running the recommended larger validation above, the authors should also include a peptide RMSD or COM distance metric, to show that the peptide identity is confident, and the peptide placement is roughly correct. These predictions are not nearly as valuable if AlphaFold is getting the right answer for the wrong reasons (i.e. high pLDDT but peptide binding to a nonCDR loop region). Eventual users of the software will likely want to make point mutations or perturb the binding regions identified by the structural predictions (as the authors do in Figure 4).

We agree with the reviewer that pLDDT is not a perfect metric, and we are following with great interest the evolving community discussion as to what metrics are most predictive of binding affinity (e.g. pAE, or pITM as a decent predictor for binding, but not affinity ranking). To our knowledge, there is not yet a consensus for the most predictive metrics for protein:protein binding nor protein:peptide binding. Intriguingly, since the antigen peptides are so small in our case, the pLDDT of the peptide residues should be mostly reporting on the confidence of the distances to neighboring protein residues.

As to the suggestion for a RMSD or COM distance metric, we agree that these are useful -with the caveat that these require a reference structure. The goal of our method is to quickly narrow down candidate linear epitopes and thereby guide experimentalists to more efficiently determine the actual binding sequence of an antibody-antigen sequence. Presumably this would not be necessary if a reference structure were known.

It may also be possible to invent a method to filter unlikely binding modes that is specific to antibodies and peptide epitopes that does not require a known reference structure, but this would be an interesting problem for subsequent study.

**Reviewer 1 (Recommendations for the Authors):**

(1) "Linear epitope" should be more precisely defined in the text. It isn't clear whether the authors hope that they can use AlphaFold to predict where on a given protein antigen an antibody will bind, or which antigenic peptide the antibody will bind to. The authors discuss both problems, and there is an important distinction between the two. If the authors are only concerned with isolated antigenic peptides, rather than linear epitopes in their full length structural contexts, they should be more precise in the introduction and discussion.

We thank the reviewer for the prompt towards higher precision. We are using the short contiguous antigen definition of “linear epitope” that depends on secondary rather than tertiary structure. The linear epitopes this paper considers are short “peptides” that form secondary structure independent of their structure in the complete folded antigen protein. We have clarified our definition of “linear epitope” in the text (lines 64-66).

(2) Line 101: "Not all portions of the antibody are critical". First, this is not consistent with the literature, particularly where computational biology is concerned.

See https://pubs.acs.org/doi/10.1021/acs.jctc.7b00080 . Second, while I largely agree with what I think the authors are trying to say (that we can largely reduce the problem to the CDR loops), this is inconsistent with what the authors later find, which is that inexplicably the VH/VL scaffold used alters results strongly.

We have adopted verbiage that should be less provocative: “Fortunately, with respect to epitope specificity, antibody constant domains are less critical than the CDR loops and the remainder of the variable domain framework regions.”

(3) Related to the above comment, do the authors have any idea why epitope prediction performance improved for the chimeric scFvs? Is this due to some stochasticity in AlphaFold? Or is there something systematic? Expanding the test dataset would again help answer this question.

We agree that future study with a larger test set could help address this intriguing result, for which we currently lack a conclusive explanation. Part of our motivation for this publication was to bring to light this unexpected result. Notably, these framework differences are not only implicated as a factor in driving AF2 performance, but also changing experimental intracellular performance as reported by our group (DOI: 10.1038/s41467-019-10846-1). We can generate a variety of hypotheses for this phenomenon. Just as MSA sub-sampling has been a popular approach to drive AF2 to sample alternative conformations, sequence recombination may be a generically effective way to generate usefully different binding predictions. However, it is difficult to discriminate between recombination inducing subtle structural tweaks that increase protein intracellular fitness and binding, from recombination causing changes to the MSA that affect the likelihood of sampling a good epitope binding conformation. It is also possible that the chimeras are more deftly predicted by AF2 due to differences in sequence representation during the training of the AF2 models (e.g. more exposure to models containing 15F11 or 2E2 structures). We attempted to deconvolute MSA differences by using single-sequence mode (Supplementary Figure 13) but this ablated performance.

(4) Figure 2: The reported consensus pLDDT scores are actually quite low here, suggesting low confidence in the result. This is in strong contrast to the reported consensus scores for mBG17. Again, a larger test dataset would help set a quantitative cutoff for where to draw the line for "trustworthy" AlphaFold predictions in antibody-peptide binding applications.

We agree that a larger dataset will be useful to begin to establish metrics and thresholds and will contribute to the aforementioned community discussion about reliable predictors of binding. Our current focus is not structure prediction per se. In the current work we are more focused on *relative* binding likelihood and increasing the efficiency of experimental epitope verification by flagging the most likely linear epitopes. Thus, while the pLDDT scores are low for Myc in Figure 2, it is remarkable (and worth reporting) that there is still useful signal in the relative variation in pLDDT. The utility of the signal variation is evident in the ability to short-list correct lead peptides via the two methods we demonstrate (consensus and per-residue max).

(5) Figure 4: if the authors are going to draw conclusions from the actual structure predictions of AlphaFold (not just the pLDDT scores), the side-chain accuracy placement should be assessed in the test dataset (RMSD or COM distance).

We agree with the reviewer that side-chain placement accuracy is important when evaluating the accuracy of AF2 structure predictions. However, here our focus was relative binding likelihood rather than structure prediction. The one case where we attempted to draw conclusions from the structure prediction was in the context of mBG17, where there is not yet an experimental reference structure. Absolutely, if we were to obtain a crystal structure for that complex, we would assess side-chain placement accuracy.

(6) Lines 493-508: I am not sure that this assessment for why AlphaFold has difficulty with antibody-antigen interactions is correct. If the authors' interpretation is correct (larger complicated structures are more challenging to move) then AlphaFold-Multimer (https://www.biorxiv.org/content/10.1101/2021.10.04.463034v2.full) wouldn't perform as well as it does. Instead, the issue is likely due to the incredibly high diversity in antibody CDR loops, which reduces the ability of the AlphaFold MSA step (which the authors show is quite critical to predictions: Figure S13) to inform structure prediction. This, coupled with the importance of side chain placement in antibody and TCR interactions, which is notoriously difficult (https://elifesciences.org/articles/90681), are likely the largest source of uncertainty in antibody-antigen interaction prediction.

We agree with the reviewer that CDR loop diversity (and associated side chain placement challenges) are a major barrier to successfully predict antibody-antigen complexes. Presumably this is true for both peptide antigens and protein antigens. Indeed, the authors of AlphaFold-multimer admit that the updated model struggles with antibody-antigen complexes, saying “As a limitation, we observe anecdotally that AlphaFold-Multimer is generally not able to predict binding of antibodies and this remains an area for future work.” The point about how loop diversity could reduce MSA quality is well taken. We have included the following thanks to the guidance of the reviewer when discussing MSA sensitivity is discussed later on in lines 570-572.:

“These challenges are presumably compounded by the incredible diversity of the CDR loops in antibodies which could decrease the useful signal from the MSA as well as drive inconsistent MSA-dependent performance”.

With respect to lines 493-508, we have also rephrased a key sentence to try to better explain that we are comparing the often-good recognition performance for short epitopes to the never-good performance when those epitopes are embedded within larger sequences. Instead of saying, “In contrast, a larger and complicated structure may be more challenging to move during the AlphaFold2 structure prediction or recycle steps.” we now say in lines 520-522 , “In contrast, embedding the epitope within a larger and more complicated structure appears to degrade the ability of AlphaFold2 to sample a comparable bound structure within the allotted recycle steps.”

(7) Related to major comment 1: Are AlphaFold predictions deterministic? That is, if you run the same peptide through the PAbFold pipeline 20 times, will you get the same pLDDT score 20 times? The lack of reproducibility may be in part due to stochasticity in AlphaFold, which the authors could actually leverage to provide more consistent results.

This is a good question that we addressed while dissecting the variable performance. When the random seed is fixed, AF2 returns the same prediction every time. After running this 10 times with a fixed seed, the mBG17 epitope was predicted with an average pLDDT of 88.94, with a standard deviation of 1.4 x 10^-14^. In contrast, when no seed is specified, AF2 did not return an *identical* result. However, the results were still remarkably consistent. Running the mBG17 epitope prediction 10 times with a different seed gave an average pLDDT of 89.24, with a standard deviation of 0.49.

(8) Related to major comment 2: The authors could use, for example, this previous survey of 1833 antibody-antigen interactions (https://www.sciencedirect.com/science/article/pii/S2001037023004725) the authors could likely pull out multiple linear epitopes to test AlphaFold's performance on antibody peptide interactions. A large number of tests are necessary for validation.

We thank the reviewer for this report of antibody-antigen interactions and will use it as a source of complexes in a future expanded study. Given the quantity and complexity of the data that we are already providing, as well as logistical challenges for compute and personnel the reviewer is asking for, we must defer this expansion to future work.

(9) Related to major comment 3: Apologies if this is too informal for a review, but this Issue on the AlphaFold GitHub may be useful: https://github.com/googledeepmind/alphafold/issues/416 .

We thank the reviewer for the suggestion – per our response above we have indeed run predictions with no templates. Since we are using local AlphaFold2 calculations with localcolabfold, the use or non-use of templates is fairly simple: including a “—templates” flag or not.

(10) Related to major comment 4: I am not sure if AlphaFold outputs by-residue secondary structure prediction by default, but I know that Phyre2 does http://www.sbg.bio.ic.ac.uk/~phyre2/html/page.cgi?id=index .

To our knowledge, AF2 does not predict secondary structure independent of the predicted tertiary structure. When we need to analyze the secondary structure we typically use the program DSSP from the tertiary structure.

(11) The documentation for this software is incomplete. The GitHub ReadMe should include complete guidelines for users with details of expected outputs, along with a thorough step-by-step walkthrough for use.

We thank the reviewer for pointing this out, but we feel that the level of detail we provide in the GitHub is sufficient for users to utilize the method described.

Stylistic comments:

(1) I do not think that the heatmaps (as in 1C, top) add much information for the reader. They are largely uniform across the y-axis (to my eyes), and the information is better conveyed by the bar and line graphs (as in 1C, middle and bottom panels).

We thank the reviewer for this feedback but elect to leave it in on the premise of more data presented is (usually) better. Including the y-axis reveals common patterns such as the lower confidence of the peptide termini, as well as the lack of some patterns that might have occurred. For example, if a subset of five contiguous residues was necessary and sufficient for local high confidence this could be visually apparent as a “staircase” in the heat map.

(2) A discussion of some of the shortcomings of other prediction-based software (lines 7177) might be useful. Why are these tools less well-equipped than AlphaFold for this problem? And if they have tried to predict antibody-antigen interactions, why have they failed?

We agree with the reviewer that a broader review of multiple methods would be interesting and useful. One challenge is that the suite of available methods is evolving rapidly, though only a subset work for multimeric systems. Some detail on deficiencies of other approaches was provided in lines 71-77 originally, although we did not go into exhaustive detail since we wanted to focus on AF2. We view using AF2 in this manner is novel and that providing additional options predict antibody epitopes will be of interest to the scientific community. We also chose AF2 because we have ample experience with it and is a software that many in the scientific community are already using and comfortable with. Additionally, AF2 provided us with a quantification parameter (pLDDT) to assess the peptides’ binding abilities. We think a future study that compares the ability of multiple emerging tools for scFv:peptide prediction will be quite interesting.

(3) Similar to the above comment, more discussion focused on why AlphaFold2 fails for antibodies (lines 126-128) might be useful for readers.

We thank the reviewer for the suggestion. The following line has been added shortly after lines 135-137:

“Another reason for selecting AF2 is to attempt to quantify its abilities the compare simple linear epitopes, since the team behind AF-multimer reported that conformational antibody complexes were difficult to predict accurately (14).”

Per earlier responses, we also added text that flags one particular possible reason for the general difficulty of predicting antibody-antigen complexes (the diversity of the CDR loops and associated MSA challenges).

(4) The first two paragraphs of the results section (lines 226-254) could likely be moved to the Methods. Additionally, details of how the scores are calculated, not just how the commands are run in python, would be useful.

Per the reviewer suggestion, we moved this section to the end of the Methods section. Also, to aid in the reader’s digestion of the analysis, the following text has been added to the Results section (lines 256-264):

“Both the ‘Simple Max’ and ‘Consensus’ methods were calculated first by parsing every pLDDT score received by every residue in the antigen sequence sliding window output structures. From the resulting data structure, the Simple Max method simply finds the maximum pLDDT value ever seen for a single residue (across all sliding windows and AF2 models). For the Consensus method, per-residue pLDDT was first averaged across the 5 AF2 models. These averages are reported in the heatmap view, and further averaged per sliding window for the bar chart below.

In principle, the strategy behind the Consensus method is to take into account agreement across the 5 AF2 models and provide insight into the confidence of entire epitopes (whole sliding windows of n=10 default) instead of disconnected, per-residue pLDDT maxima.”

(5) Figure 1 would be more useful if you could differentiate specifically how the Consensus and Simple Max scoring is different. Providing examples for how and why the top 5 peptide hits can change (quite significantly) using both methods would greatly help readers understand what is going on.

Per the reviewer suggestion, we have added text to discuss the variable hit selection that results from the two scoring metrics. The new text (lines 264-271) adds onto the added text block immediately above:

“Having two scoring metrics is useful because the selection of predicted hits can differ. As shown in Figure 2, part of the Myc epitope makes it into the top 5 peptides when selection is based on summing per-residue maximum pLDDT (despite there being no requirement that these values originate in the same physical prediction). In contrast, a Consensus method score more directly reports on a specific sliding window, and the strength of the highest confidence peptides is more directly revealed with superior signal to noise as shown in Figure 3. Variability in the ranking of top hits between the two methods arises from the fundamental difference in strategy (peptide-centric or residue-centric scoring) as well as close competition between the raw AF2 confidence in the known peptide and competing decoy sequences.”

(6) Hopefully the reproducibility issue is alleviated, but if not the discussion of it (lines 523554) should be moved to the supplement or an appendix.

The ability of the original AF2 model to predict protein-protein complexes was an emergent behavior, and then an explicit training goal for AF2.multimer. In this vein, the ability to predict scFv:peptide complexes is also an emergent capability of these models. It is our hope that by highlighting this capacity, as well as the high level of sensitivity, that this capability will be enhanced and not degraded in future models/algorithms (both general and specialized). In this regard, with an eye towards progress, we think it is actually important to put this issue in the scientific foreground rather than the background. When it comes to improving machine learning methods negative results are also exceedingly important.

**Reviewer 2 (Recommendations for the Author):**

- Line 113, page 3 - the structures of the novel scFv chimeras can be rapidly and confidently be predicted by AlphaFold2 to the structures of the novel scFv chimeras can be rapidly and confidently predicted by AlphaFold2.

The superfluous “be” was removed from the text.

- Line 276 and 278 page 9 - peptide sequences QKLSEEDLL and EQKLSEEDL in the text are different from the sequences reported in Figures 1 and 2 (QKLISEEDLL and EQKLISEEDL). Please check throughout the manuscript and also in the Figure caption (as in Figure 2).

These changes were made throughout the text.

- I would include how you calculate the pLDDT score for both Simple Max approach and Consensus analysis.

Good suggestion, this should be covered via the additions noted above.
